# Sympatry and interference of divergent *Microbotryum* pathogen species

**DOI:** 10.1002/ece3.5140

**Published:** 2019-04-12

**Authors:** Michael E. Hood, Janis Antonovics, Monroe Wolf, Zachariah L. Stern, Tatiana Giraud, Jessica L. Abbate

**Affiliations:** ^1^ Department of Biology Amherst College Amherst Massachusetts; ^2^ Department of Biology University of Virginia Charlottesville Virginia; ^3^ Ecologie Systematique et Evolution, Univ. Paris‐Sud, CNRS, AgroParisTech Université Paris Saclay Orsay France; ^4^ INRA ‐ UMR 1062 CBGP (INRA, IRD, CIRAD, Montpellier SupAgro) Montferrier‐sur‐Lez France

**Keywords:** anther‐smut, disease distributions, host‐shift, pathogen competition, pathogen competitive interference, systemic acquired resistance

## Abstract

The impact of infectious diseases in natural ecosystems is strongly influenced by the degree of pathogen specialization and by the local assemblies of potential host species. This study investigated anther‐smut disease, caused by fungi in the genus *Microbotryum*, among natural populations of plants in the Caryophyllaceae. A broad geographic survey focused on sites of the disease on multiple host species in sympatry. Analysis of molecular identities for the pathogens revealed that sympatric disease was most often due to co‐occurrence of distinct, host‐specific anther‐smut fungi, rather than localized cross‐species disease transmission. Flowers from sympatric populations showed that the *Microbotryum* spores were frequently moved between host species. Experimental inoculations to simulate cross‐species exposure to the pathogens in these plant communities showed that the anther‐smut pathogen was less able to cause disease on its regular host when following exposure of the plants to incompatible pathogens from another host species. These results indicate that multi‐host/multi‐pathogen communities are common in this system and they involve a previously hidden mechanism of interference between *Microbotryum* fungi, which likely affects both pathogen and host distributions.

## 1 INTRODUCTION

Infectious diseases have a major influence on natural communities, and both positive and negative effects on biodiversity have been recorded (Benítez, Hersh, Vilgalys, & Clark, 2013; Johnson, Roode, & Fenton, 2015; Mordecai, 2011; Seabloom et al., 2015; Young, Parker, Gilbert, Sofiea Guerra, & Nunn, 2017). It is generally expected that diseases with high host specificity should increase diversity by limiting the ecological extent of any one host species, while generalist diseases might have negative overall effects as transmission is not dependent on the abundance of any one host type. However, in many cases uncertainty remains about the specialization of disease interactions, resulting from the lack of morphological traits that readily distinguish related pathogen species. In some cases, observation of a disease affecting multiple, sympatric host species has provided opportunities to study transient host‐shifts, disease emergence, and pathogen diversification (Antonovics, Hood, & Partain, 2002; Biek et al., 2012; Craft, Volz, Packer, & Meyers, 2009; Davies & Pedersen, 2008; Fournier & Giraud, 2008; Luis et al., 2015). In comparison, we know less about the occurrence of disease in a mixed‐host community when it is the result of each host carrying their own specialized pathogens. If these specialized pathogens do show some level of cross‐species movement, we still may expect possible consequences varying from cross‐protection (e.g., due to systemic acquired resistance or pathogen competitive interference), to facilitation (e.g., due to increased susceptibility of previously infected hosts), or to pathogen hybridization (e.g., Shykoff, Meyhöfer, & Bucheli, 1999; Newcombe, Stirling, McDonald, & Bradshaw, 2000; Ricklefs, 2010; Gladieux et al., 2011; Kulma, Low, Bensch, & Qvarnström, 2013; Ellis et al., 2015).

**Figure 1:**
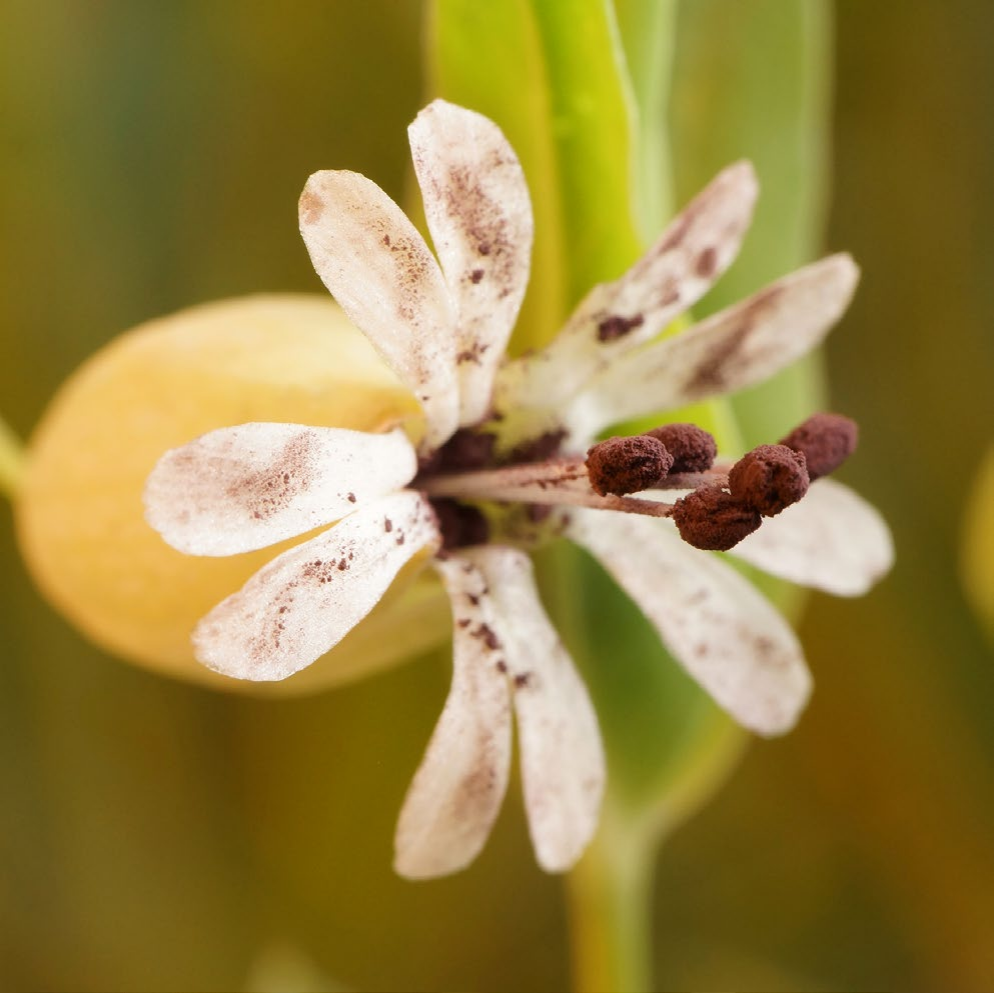
Anther‐smut disease caused by fungi in the genus *Microbotryum*, here shown in *Silene vulgaris*. The pollen is replaced by masses of dark pathogen spores

Anther‐smut disease, caused by fungi in the genus *Microbotryum*, is one of the most studied host–pathogen associations in natural systems (Figure 1). This disease has been used as a model for pathogen speciation, pollinator‐mediated dispersal, multiple infection, biological invasion, and competition (Abbate & Antonovics, 2014; Bruns, Antonovics, & Hood, 2018; Fontaine, Gladieux, Hood, & Giraud, 2013; Giraud, Gladieux, & Gavrilets, 2010; Gold, Giraud, & Hood, 2009; Kemler et al., 2013; Le Gac, Hood, Fournier, & Giraud, 2007; Vercken et al., 2010). Research has shown that the fungi formerly grouped under the *Microbotryum violaceum* epithet represent a large species complex, consisting of many independent lineages, each specific (i.e., endemic) to only one or a very small number of host species (Kemler et al., 2013; Le Gac et al., 2007; Lutz et al., 2005; Piątek, Lutz, & Kemler, 2013; de Vienne, Hood, & Giraud, 2009). However, in marked contrast to the strong host specificity generally seen in those phylogenetic studies, several instances of cross‐species disease transmission have been observed in nature (Antonovics et al., 2002; Gladieux et al., 2011; López‐Villavicencio et al., 2007; Refrégier et al., 2008; Tyson, Antonovics, & Bruns, 2018). Cross‐inoculations under experimental conditions further support the potential for host‐shifts in some nonendemic host–pathogen combinations (Biere & Honders, 1996; Van Putten, Biere, & Damme, 2005; Shykoff et al., 1999; Sloan, Giraud, & Hood, 2008; de Vienne et al., 2009). In particular, the widespread European native host *Silene vulgaris* becomes naturally and experimentally diseased through exposure to *Microbotryum* from the host *Silene latifolia* (Antonovics et al., 2002; Hood, Antonovics, & Heishman, 2003; de Vienne et al., 2009), although the generality of these findings to other host species are not known.

In the current study, we analyzed DNA sequence identities of *Microbotryum* samples that were collected from a large number of natural populations, focusing on localities where multiple diseased host species were growing together. Our first goal was to determine whether localities with the disease on multiple host species represented sites of cross‐species transmission or the sympatry of divergent pathogen lineages each specialized to their co‐occurring host species. Our second goal was to investigate the movement of *Microbotryum* spores between sympatric host species by microscopic examination of spore deposition on flowers. Our third goal addressed a potential consequence of sympatry of pathogen lineages, experimentally investigating the potential for interference (direct or indirect) among divergently specialized pathogens, noting that the topic of hybridization in *Microbotryum* has been addressed elsewhere (Abbate et al., 2018; Gladieux et al., 2011; Petit et al., 2017; Shykoff et al., 1999). Specifically, we asked whether the ability of the endemic pathogen of *S. vulgaris* to infect its natural host was altered by prior exposure of plants with nonendemic pathogens from other host species. This study helps to reveal a complex source of interactions between relatively specialized pathogens on multiple host species and their potential impact on the occurrence of disease in natural populations.

## 2 MATERIALS AND METHODS

### 2.1 Study system

*Microbotryum* is a basidiomycete genus of anther‐smut fungi in the Pucciniomycotina subphylum. Taxonomic revisions of the *M. violaceum* (formerly *Ustilago violacea*) species complex are ongoing (e.g., Le Gac et al., 2007; Denchev, Giraud, & Hood, 2009; Piątek et al., 2013; Ziegler, Lutz, Piątek, & Piątek, 2018), and all samples will be referred to here by the genus *Microbotryum* and the host‐of‐origin (e.g., *Mv – S. latifolia*); where available, Latin binomials are given in Supporting Information Table S1. The disease is spread by insect pollinators that transmit the fungal spores produced in the anthers of infected plants (Figure 1). Transmission between host species is favored by shared flowering times and generalist pollinator guilds (Shykoff et al., 1999; Tang et al., 2019). However, infection through vegetative parts of juvenile nonflowering plants can also occur (Alexander & Antonovics, 1988; Alexander & Maltby, 1990; Bruns, Antonovics, Carasso, & Hood, 2017).

As with many diseases, the anther‐smut life history plays a role in shaping where the pathogen is found in nature. Anther‐smut fungi in the genus *Microbotryum* are obligate, biotrophic pathogens of perennial plants mainly in the Caryophyllaceae (Hood et al., 2010; Thrall, Biere, & Antonovics, 1993), where infections cause host sterility, are systemic and persistent, and have the potential to competitively exclude subsequent invasion by other *Microbotryum* pathogens (Gold et al., 2009; Hood, 2003; López‐Villavicencio et al., 2011). There are many host species for anther smut with broad and overlapping geographic distributions (Hitchcock & Maguire, 1847; Hood et al., 2010), and disease incidence can be remarkably high in some host species. For example, the typical proportions of disease within host populations are between 0.10 and 0.30 for *S. latifolia* (Antonovics, 2004), ca. 0.30 for *S. vuglaris* in upper elevations (Abbate & Antonovics, 2014), or even >0.50 for *Dianthus furcatus* or *Dianthus pavonius* (Bruns et al., 2018). Therefore, the potential for frequent contact between *Microbotryum* species on separate host species is high.

### 2.2 Field specificity on sympatric host species

The fungus was sampled from natural populations as the spore contents of single diseased flowers, which are expected to contain one fungal genotype (Garber & Ruddat, 2002; Hood, 2003; Van Putten et al., 2005). Flowers were sampled at the bud stage whenever possible (i.e., to avoid possible contamination by pollinator visits) and stored under desiccation prior to use.

**Figure 2:**
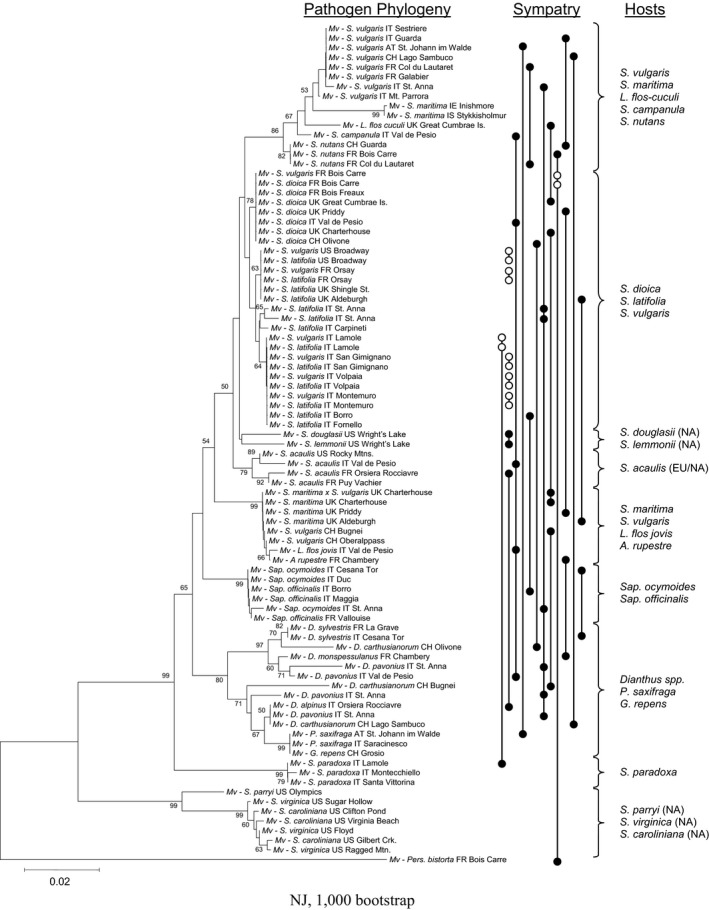
The left side shows the neighbor‐joining tree of the *Microbotryum* samples collected from multiple host species and localities. Collection sites where the disease was on more than one host species are indicated on the right side by vertically arranged dots, connected by vertical bars. Filled dots indicate sympatry of lineages found only on their respective (specific) host species. Open dots indicate instances of a single pathogen lineage infecting multiple sympatric host species. Brackets indicate groups of host species sharing phylogenetically similar pathogens and their source: samples are from Europe (EU) unless otherwise indicated as being from North America (NA)

Sampling was carried out over several years during the course of fieldwork in many geographic regions and included diseased plants in the genera *Silene*, *Lychnis*, *Atocion*, *Dianthus*, *Saponaria*, *Gypsophila*, and *Petrorhagia* (Figure 2). Sympatry of *Microbotryum* on two or more host species was defined as when the disease was found on another host within easy search distance (ca. 100 m) of the first‐found diseased host. A collection of samples where the disease was on only one host species was also included to increase the inference of host‐specific pathogen lineages when occurring across localities.

To assessing disease transmission among sympatric host species within localities, we used DNA sequence identity for the pathogens' internal transcribed spacer (ITS) region of the nuclear rDNA. While ITS sequences have utility in resolving fungal lineages (Schoch et al., 2012; but see Hart et al., 2015), phylogenetic insights were secondary and need to be interpreted cautiously due to the use of a restricted amount of DNA sequence length and variation in statistical support among nodes. Nevertheless, to display the ITS sequence variation, neighbor‐joining analysis (Jukes‐Cantor distances) was performed using the MEGA software (Kumar, Tamura, Jakobsen, & Nei, 2001) with 1,000 bootstrap replications. The smut fungus infecting *Persicaria bistorta* (Syn: *Polygonum bistorta*) in the Polygonaceae was used as an outgroup because it is from outside the *Microbotryum* clade causing anther‐smut disease on the Caryophyllaceae (Almaraz, Roux, Maumont, & Durrieu, 2002; Kemler, Lutz, Göker, Oberwinkler, & Begerow, 2009).

To prepare cultures for DNA extraction, the spores (i.e., teliospores, diploid) were plated on potato dextrose agar at 25°C. Upon germination, the fungus undergoes meiosis, and the resulting haploid sporidia can be grown as yeast‐like cultures. Colonies derived from single haploid sporidia were obtained by microdissection or by dilution plating. All sporidial cultures were stored under desiccation at −20°C.

DNA was extracted from sporidial cultures using the DNeasy Plant Mini Kit (Qiagen). PCR was used to amplify the ITS region (primers: ITS1 and ITS4 from White, Bruns, Lee, & Taylor, 1990). PCR products were sequenced using Sanger dye termination methods. For some sequences, where the chromatograms contained discrete regions of basepair ambiguities, individual ITS amplicons were cloned using the TA Cloning Kit (Invitrogen) and sequenced. Sequence alignments were prepared using ClustalW (www.ebi.ac.uk/clustalw/), with minor adjustments performed manually. DNA sequences are available in GenBank under accession numbers KY084313–KY084399.

### 2.3 Spore dispersal among sympatric host species

The potential for movement of *Microbotryum* spores from the diseased flowers of one host species to another sympatric host species was assessed in natural mixed plant communities. Using sites with two host species, but where only one was diseased, healthy flowers of the unaffected host species were collected in sealed coin envelopes. Distance to nearest disease plant of the other host species was recorded. Various host species combinations were examined (Table 1), with the co‐occurrence of healthy *S. vulgaris* with diseased *Saponaria officinalis* being the most common. Samples were collected within the census region of Bruns et al. (2018) in the western Alps, from 21 localities where co‐occurring hosts were encountered.

**Table 1:** Movement of *Microbotryum* spores among sympatric host species

Diseased source species	Healthy target species	Number of target flowers examined	Number of target flowers containing spores
*Dianthus pavonius*	*Silene campanula*	1	1
*Dianthus pavonius*	*Arenaria sp*.	2	2
*Knautia arvensis*	*Dianthus pavonius*	3	3
*Silene acaulis*	*Silene campanula*	1	1
*Saponaria officinalis*	*Silene latifolia*	12	11
*Saponaria officinalis*	*Silene vulgaris*	50	49
*Silene campanula*	*Silene pusilla*	6	5
*Silene campanula*	*Silene saxifraga*	1	1
*Silene latifolia*	*Silene vulgaris*	1	1
*Silene pusilla*	*Silene campanula*	3	3
*Silene saxifraga*	*Dianthus pavonius*	1	1
*Silene vulgaris*	*Saponaria officinalis*	1	1
Total		82	79

For spore counting, the flowers were rehydrated in 2 ml microcentrifuge tubes containing 0.7 ml of 0.2% Triton X‐100 and vortexed vigorously. The flower was removed, and the suspended contents were concentrated by centrifugation and removal of 90% of the supernatant. Microscopic quantification of spores was performed as two full‐width transects of 10 μl of concentrated flower contents spread under a 22 × 22 mm coverslip for each of two replicates per sample. For calibration, we applied spore concentrations to healthy flowers of *S. vulgaris*, ranging from 400,000 to 40 spores per flower, in a ten‐fold dilution series, and followed the same protocol as for field‐collected flowers. As samples from healthy *S. vulgaris* with diseased *Sa. officinalis* were numerous, data from this combination of host species were further analyzed for an effect of proximity to diseased plants upon heterospecific deposition of *Microbotryum* spores, using GLM procedures in SPSS v19 (SPSS, Chicago, Illinois, USA).

### 2.4 Consequences of cross‐species pathogen exposure

To assess whether the ability of *Microbotryum* to cause disease on its host‐of‐origin (i.e., endemic host–pathogen combination) was affected by prior exposure of the host to *Microbotryum* obtained from another host (i.e., nonendemic pathogen), experimental inoculation of *S. vulgaris* was designed with treatments including each of three nonendemic pathogens. An initial nonendemic inoculation was followed by inoculation with an endemic pathogen from *S. vulgaris*, *Microbotryum silenes‐inflatae*. The time interval between nonendemic and endemic inoculations was varied as second treatments of 7 or 10 days, and the proportions of plants diseased by the endemic pathogen for all treatments were compared to paired sets of plants initially treated with water as a control followed by the endemic pathogen at the same time intervals. After low disease rates in the first run of the experiment, which is usual for this host species (Abbate & Antonovics, 2014), an independent second run of the experiment was performed and combined with the first (following test of run significance, below). All treatments totaled between 107 and 140 plants surviving to flower that were randomized for positions in the greenhouse.

Seeds of *S. vulgaris* were collected in natural populations in Berkshire County, Massachusetts. For use as nonendemic pathogens for this experiment, *Microbotryum* samples were obtained from the following hosts in natural populations: *S. latifolia* (*M. lychnidis‐dioicae*, abbreviated MvSl), *Silene paradoxa* (*Microbotryum* sp., abbreviated MvSp), and *S. officinalis* (*M. saponariae*, abbreviated MvSap). While the inoculum samples were not from sympatric populations with *S. vulgaris*, the pathogens were chosen because of the common co‐occurrence of their host species with the widespread *S. vulgaris* distribution in Europe and their availability in our prior collections. Also, the pathogens from *S. paradoxa* and *Sa. officinalis* were previously shown to be unable to cause anther‐smut disease on *S. vulgaris* under experimental inoculation (de Vienne et al., 2009), while the pathogen from *S. latifolia* was the source of host‐shift disease observed in the current and prior studies (Antonovics et al., 2002; Hood et al., 2003).

Seeds of *S. vulgaris* were surface sterilized (2 min in 2% sodium hypochlorite, 20% ethyl alcohol, and 0.2% Triton X‐100) and then germinated in soil in 3.81‐cm Conetainers (Stuewe and Sons, Corvallis, OR) under greenhouse conditions. When the cotyledons had separated, 4 μl of a 500 spore/μl suspension was placed on the apical meristem. For each treatment, a paired set of equal number of plants received either the initial pathogen inoculum or water control. Plants were then covered with plastic wrap to maintain humidity without watering at 15°C. After 7 or after 10 days, these plants were inoculated with the pathogen endemic to *S. vulgaris*.

Disease status of plants (i.e., smut spores in the anthers) was scored at flowering. *Microbotryum* infections producing disease in the experimental plants were confirmed to be *M. silenes‐inflatae* by phenotypic variation in pathogen culture morphologies; *M. silenes‐inflatae* produces colonies in vitro with markedly reduced growth compared to the other species (as assessed in Gold et al., 2009).

Proportions of plants that became diseased by the pathogen endemic to *S. vulgaris* were compared for each nonendemic pathogen treatment to the proportion in their water control treated plants. The effects of host exposure to nonendemic pathogens on the ability of the endemic pathogen to cause disease were assessed using GLM procedures in SPSS v19 following arcsin square‐root data transformation of the proportions.

## 3 RESULTS

### 3.1 Field specificity on sympatric host species

Samples of *Microbotryum* were obtained in North America and in Europe from 24 localities with anther‐smut disease on more than one host species and 27 localities containing disease on only one host species. Analysis of the DNA sequences revealed that *Microbotryum* consisted largely of ITS variants specific to one or a very few host species (Figure 2). Moreover, in 19 of 24 localities with multiple diseased host species, the co‐occurrence of diseases resulted from the sympatry of divergent lineages of the pathogen that were particular to their hosts‐of‐origin.

The exceptions to this trend of disease on two host species being the result of sympatry between host‐specific *Microbotryum* lineages were several samples collected from *S. vulgaris* that resulted from cross‐species disease transmission. Seven localities were found where *Microbotryum* on *S. vulgaris* was identical in ITS sequence to the sympatric pathogen known to be specialized on *S. latifolia* or *Silene dioica*; two of these localities also contained other sympatric *Microbotryum* pathogens (Figure 2). These instances of cross‐species transmission were likely independent because they were found to be consistent with slight regional differences in ITS sequence among *Microbotryum* from *S. latifolia* or were from different geographic locations. Despite evidence from prior reports of cross‐species disease transmission by *Microbotryum* between *S. dioica* and *S. latifolia* (Gladieux et al., 2011), sympatric diseased populations of these two host species were not obtained in this study (Figure 2).

In some sections of the tree, *Microbotryum* from closely related host species were not seen to be host‐specific (Figure 2). This was the case for lineages occurring on various *Dianthus* species, similar to prior reports (e.g., Le Gac et al., 2007; Kemler et al., 2013; Petit et al., 2017). Large genetic distances in ITS sequences were observed among samples of *Microbotryum* from *Dianthus carthusianorum*, and similarly from *D. pavonius* (Figure 2), in agreement with multiple, less specialized pathogen species infecting *Dianthus* hosts (Le Gac et al., 2007; Petit et al., 2017). Moreover, ITS of *Microbotryum* from several *Dianthus* species carried discrete regions of basepair ambiguities in the chromatograms, indicative of intragenomic heterogeneity in this multi‐copy gene that is consistent with prior reports on hybridization (Petit et al., 2017). Cloning of PCR products from one such sample revealed distinct ITS amplicons, which each fell among other *Microbotryum* samples from *Dianthus* species.

Samples of *Microbotryum* from *S. caroliniana* and *S. virginica* were interdigitated on the tree, but this may result from insufficient resolution to detect distinct *Microbotryum* lineages. Samples of *Microbotryum* from *Sa. ocymoides* and *Sa. officinalis* were also intermingled across a large geographic range in agreement with studies of microsatellite markers that could not differentiate host‐specialized lineages on these two hosts (Fortuna et al., 2018).

Comparison of samples from Europe and North America revealed that divergent lineages of *Microbotryum* broadly overlapped in geographic range. Some samples native to North America had DNA sequences similar to *Microbotryum* from the previously described European clade of *Microbotryum* (Lutz et al., 2005). Specifically, *Microbotryum* from *S. douglasii* and *S. lemmonii* on the west coast of North America and from *S. acaulis* in the Rocky Mountains clustered with the remaining samples from Europe. However, *Microbotryum* from *S. paryii*, also on the west coast of North America, more closely resembled the fungus from *S. caroliniana* and *S. virginica* from the east coast of the continent.

### 3.2 Spore dispersal among sympatric host species

**Figure 3:**
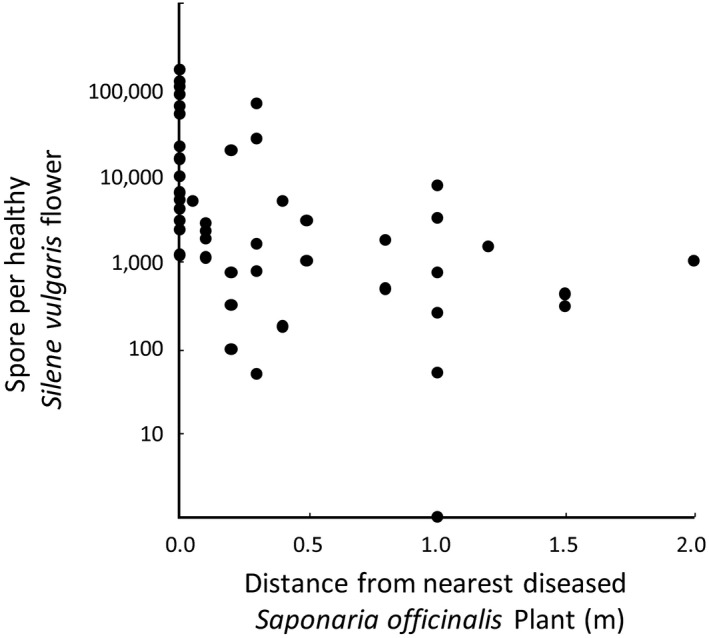
Movement of *Microbotryum* spores between the host species *Saponaria officinalis* and *Silene vulgaris*. Samples were from sites containing diseased *Sa. officinalis* and healthy *S. vulgaris*. Spores per flower were estimated based upon a dilution factor and standardization with experimental flowers containing spores applied at known concentrations

*Microbotryum* spores are readily found on the flowers of nondiseased species when they occur in mixed‐host communities. Examination of flowers from the host species that was only healthy in a locality (target flowers) but co‐occurring with another diseased host species (disease sources) revealed cross‐species spore deposition in 96% of target flowers (Table 1). Additionally, in the target:source pair of healthy *S. vulgaris* in sympatry with disease on *Sa. officinalis*, the distance between the healthy host species and a heterospecific source of *Microbotryum* spores was significantly negatively related to the amount of spores deposited (Figure 3; SPSS linear regression, 2‐tailed *p*‐value = 0.010, *y* = −25585*x* + 29307, *R* ^2^ = 0.13).

### 3.3 Consequences of cross‐species pathogen exposure

**Figure 4:**
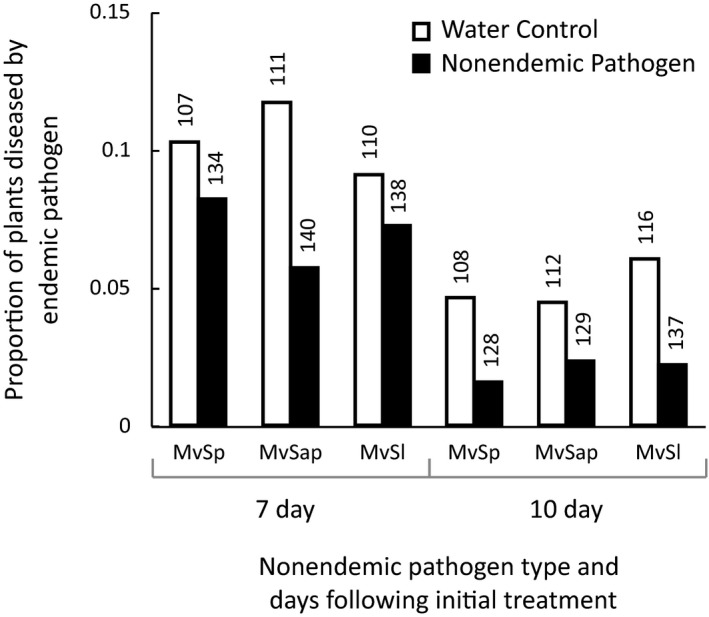
Reduced disease of *Silene vulgaris* by endemic pathogen, *Microbotryum silenes‐inflatae*, following exposure of the host to nonendemic *Microbotryum* lineages. Proportions of plants diseased by *M. silenes‐inflatae* are shown for plants treated with either a nonendemic pathogen (black columns) or water (white columns) prior to the inoculation with the pathogen endemic to *S. vulgaris*. Numbers represent sample size. Data are grouped by the nonendemic pathogen type for the initial inoculation and their paired water controls and by the separation time between the initial treatment and inoculation with the endemic *M. silenes‐inflatae*. *Microbotryum* from other host species are abbreviated by the *Mv* followed by the host initials as indicated in the text

The experimental prior exposure of plants to *Microbotryum* pathogens specialized to another host species negatively affected the ability of the endemic pathogen, *M. silenes‐inflatae*, to subsequently cause disease on its natural host, *S. vulgaris*. The proportions of plants that became diseased were consistently lower when plants received prior inoculation with a nonendemic pathogen in comparison with paired sets of plants having received only water prior to the inoculation with the endemic *M. silenes‐inflatae* (Figure 4; prior inoculum vs. water control treatments *F*_1,6_ = 28.606, *p*‐value = 0.003). Proportions of plants that became diseased by *M. silenes‐inflatae* were also lower when the inoculum was applied to older seedlings (i.e., at 10 days vs. 7 days after the initial treatment; *F*_1,6_ = 74.07, *p*‐value < 0.001). The nonendemic pathogen type, replicate inoculation series, and interaction terms were each nonsignificant.

Characterization of the pathogen on diseased experimental plants showed that no disease was produced by the preinoculation with *Microbotryum* from *S. paradoxa* or *Sa. officinalis*. However, three percent of *S. vulgaris* plants initially preinoculated with the *Microbotryum* lineage from *S. latifolia* became diseased with this nonendemic pathogen. Even though the statistical test above only considered the proportions of plants diseased by the endemic *M. silenes‐inflatae* pathogen, successful infection caused by the nonendemic *S. latifolia* pathogen may have affected the overall results. However, analysis based on only the nonendemic pathogens from *S. paradoxa* and *Sa. officinalis* still showed significantly reduced infection by the endemic *M. silenes‐inflatae* pathogen (prior inoculum vs. water control treatments *F*_1,4_ = 16.233, *p*‐value = 0.027).

## 4 DISCUSSION

In a broadly distributed multi‐host/multi‐pathogen system, this study revealed the co‐occurrence of anther‐smut diseases on sympatric host species and significant pathogen interactions that may impact disease and host distributions in natural populations. In a very early observation of disease ecology, Schröter (1877) suggested that host sympatry can be used to assess pathogen specialization, saying of anther smut that “*Ustilago violacea* [=*Microbotryum*] has been found on so many plants of the *Melandrium* [=*Silene*] family that one is tempted to assume that it lets itself be transferred to all its representatives,” while “very often one finds large stretches [of roadside] covered with *Saponaria*, their blossoms stricken with *Ustilago*, though in between are completely healthy stems of *M. album*, [=*S. latifolia*] although this plant is often enough infected with the fungus.” Restriction of the pathogen to one host while in sympatry with other potential host species is indicative of parasitic specialization, as Schröter suggests. Host species of *Microbotryum* have broadly overlapping geographic ranges (Hood et al., 2010), and many hosts can be found growing intermixed. Flowering times are often fully or partially concurrent, and pollinator guilds generally lack the strict specificity to prevent *Microbotryum* spores from being vectored between host species (e.g., this study, and Goulson & Jerrim, 1997; Shykoff et al., 1999; Jürgens, 2006; Tang et al., 2019; but see Van Putten, Elzinga, & Biere, 2007). Therefore, despite ample opportunity for host‐shifts to occur, the apparent field specificity of *Microbotryum* likely reflects trade‐offs in performance on particular plant species, as seen from experimental cross‐species inoculation (e.g., de Vienne et al., 2009; Sloan et al., 2008).

Our study shows that the alternative to Schröter's scenario, that is, the presence of anther smut on multiple sympatric host species, was rarely indicative of cross‐species transmission and a breakdown in host specificity. The great majority of cases were co‐occurrence of divergent *Microbotryum* lineages, each particular to one host species within the local plant community. It remains possible that our sampling may have missed some cross‐species disease transmission in localities where multiple *Microbotryum* lineages co‐occurred on different host species. However, even in cases where ecological circumstances were suggestive of host‐shifts (e.g., high frequency of disease on one host with very rare disease on a second intermixed host, such as involving *S. vulgaris* and *S. nutans* at Col du Lautaret; data not shown), sympatry of specialized *Microbotryum* lineages was observed. Our sampling was inadequate to assess probabilities of co‐occurrence based on disease incidence, but Hood et al. (2010) observed a positive relationship between regions of high host species richness and those host species with the highest within‐species prevalence of anther‐smut disease. Thus, where host sympatries are frequent, the probabilities of disease occurrence within host species are also high and could positively contribute to the type of multi‐pathogen/multi‐host assemblies observed here.

The co‐occurrence of multiple pathogen and multiple host species is reported to yield complex interactions and interference among pathogens that affect their community structure (Fenton, Streicker, Petchey, & Pedersen, 2015; Halliday, Heckman, Wilfahrt, & Mitchell, 2019; Johnson et al., 2015; Parker & Gilbert, 2018; Seabloom et al., 2015). We showed that cross‐species movement of *Microbotryum* spores is very common in mixed‐host communities and that prior exposure to incompatible, nonendemic pathogens reduced the ability of the adapted pathogen to cause disease in its host. A predicted impact of cross‐species spore movement on the *Microbotryum* community may be to limit the occurrence of multi‐host/multi‐pathogen assemblies. The mechanisms would be analogous to the “inhibitory host” model of Holt, Dobson, Begon, Bowers, and Schauber (2003). This model describes the situation where a second host offers no contribution to a pathogen's reproduction but diminishes a pathogen's persistence on the endemic host species, either because the second host serves as an inoculum sink or reduces visitation rates in the case of vector‐transmitted diseases (i.e., a dilution effect; Ostfeld & Keesing, 2000). In *Microbotryum*, however, our results suggest the inhibitory effect may be mediated by a “spill‐over” type of cross‐protection, such that *Microbotryum* pathogens may be less able to invade or to be maintained at as high a prevalence in host populations that are sympatric with anther‐smut disease on other host species. Whether the protection is long‐lasting, and whether it is due to competitive exclusion by asymptomatic infections or inducible host resistance mechanisms, is not yet known. Anther‐smut inoculation has been shown to induce some changes to host growth even in the absence of symptoms at flowering (Antonovics et al., 2018), but the possibility of persistent asymptomatic infections has not been investigated. Intrahost competitive exclusion may also be important and is well documented among *Microbotryum* strains, including within‐ and among‐species interactions (Fortuna et al., 2018; Gold et al., 2009; Hood, 2003). Studies have indicated heritable variation in anther‐smut disease resistance (Alexander & Maltby, 1990; Cafuir, Antonovics, & Hood, 2007; Carlsson‐Granér & Pettersson, 2005; Chung, Petit, Antonovics, Pedersen, & Hood, 2012), but the mechanisms of resistance and whether it is inducible have not been determined.

While such cross‐species protection can potentially limit local pathogen diversity, the effects on the host diversity might be in the opposite direction. Where a diseased host is common, introduction of a competing host species would be facilitated because the diseased host (acting as an “inhibitory host”) protects the introduced host against pathogen infection. In this way, the disease may contribute positively to host diversity, in a manner enhancing the density‐dependent and species‐specific feedbacks of the Janzen–Connell effect (Comita et al., 2014; Connell, 1970; Janzen, 1970). More extensive field surveys combined with experimental studies would be needed to directly investigate *Microbotryum* co‐occurrence and its impact on host and pathogen assemblages.

Exceptions to the general pattern of host specificity of *Microbotryum* included a number of cross‐species transmissions to *S. vulgaris* from either sympatric *S. latifolia* or *S. dioica*. Such host‐shifts to *S. vulgaris* have been confirmed by other genetic approaches and experimental studies (Antonovics et al., 2002; Hood et al., 2003; de Vienne et al., 2009). It remains to be determined whether *S. vulgaris* is predisposed to receiving host‐shifts due to its susceptibility or whether the ecology of being weedy and very widespread creates more opportunities for host‐shifts to occur and to be observed. Moreover, in the current and previous studies (Abbate & Antonovics, 2014; Abbate et al., 2018; Bucheli, Gautschi, & Shykoff, 2000; Le Gac et al., 2007), *S. vulgaris* from high elevations (>ca. 1,300 m) was found infected by multiple distinct, endemic, and apparently self‐sustaining lineages of *Microbotryum*, which is also consistent with a greater propensity for host‐shifts and perhaps new disease emergence onto this species. Additional exceptions to host specificity include historic host‐shifts that have been inferred from the incongruence of the host and pathogen phylogenies (Refrégier et al., 2008). Also, in the current study and previous ones (Kemler et al., 2013; Le Gac et al., 2007; Petit et al., 2017), sequence variation indicated that several lineages of *Microbotryum* from *Dianthus* can share host species of this genus, suggesting less strict pathogen specificity on this recently radiated plant genus (Valente, Savolainen, & Vargas, 2010).

At a broader scale, this study supported patterns in the geographic overlap of *Microbotryum* lineages, including samples from European and North American host species. It was previously suggested that North America contains *Microbotryum* lineages that are highly divergent from those found in Europe (Freeman, Kellye Duong, Shi, Hughes, & Perlin, 2002; Hood, Rocha, 0. J., & Antonovics, J., 2001). However, on the west coast of North America occur members of both the previously described North American clade (i.e., *Microbotryum* from *S. parryi*) and members of the European clade (i.e., *Microbotryum* from *S. lemmonii* and *S. douglasii*; see also Lutz et al., 2005). It is remarkable that such great variation in *Microbotryum* would be found in North America because the history of the genus *Silene* has a Eurasian origin followed by migration with reduced species diversity into the Americas via the Beringian region (Popp, Erixon, Eggens, & Oxelman, 2005; Popp & Oxelman, 2007). Further studies (e.g., incorporating genomic‐scale data; Branco et al., 2018) are warranted to address the large‐scale phylogeographic diversification of *Microbotryum*, and the potential impact on the co‐occurrence and interactions of pathogen lineages.

In summary, this study confirms that the host specificity seen in broad‐scale phylogenetic and experimental inoculation studies of *Microbotryum* generally reflects specialization at an ecological level and that such specialization holds true even when host species are in close sympatry. This expectation needs to be tested in any particular case, as there are exceptions, especially in hosts such as *S. vulgaris* or *Dianthus* species. However, even in a community assemblage where there is apparent host specificity, cross‐species exposure to multiple pathogen lineages is likely to occur, with consequences that may be overt or may be “cryptic,” yet still may influence dynamics of the species assemblage. This study identified one such mechanism—inhibition of endemic pathogens by prior exposure to nonendemic ones—but further effects on occurrence and distribution of the multi‐host/multi‐pathogen communities are likely and deserve further investigation.

## CONFLICT OF INTEREST

None declared.

## AUTHOR CONTRIBUTIONS

Hood and Antonovics conceptualized the project. Hood, Antonovics, Abbate, and Stern contributed to field collection and their assessment. Hood and Wolf contributed experimental studies. Hood, Antonovics, and Giraud wrote and revised the manuscript, with contribution also from Wolf and Stern.

## Data Availability

DNA sequences: GenBank accession numbers KY084313–KY084399. Sample locations: Dryad https://doi.org/10.5061/dryad.5g8h6f6.
