# Supplementary material for: Sympatry and interference of divergent Microbotryum pathogen species
Source: Ecol Evol. 2019 Apr 12;9(9):5457–67. doi: 10.1002/ece3.5140 (PMC6509394; doi:10.1002/ece3.5140)
Supplement: Supplementary file 1 [file ECE3-9-5457-s001.docx]

| **Table S1. Identification of *Microbotryum* samples used in assessing sympatry of host-specific lineages** | | | | |
| --- | --- | --- | --- | --- |
|  |  |  |  |  |
| **Name in Phylogeny** | **Latin Binomial** | **Country** | **Local** | **NCBI Accession** |
| *Mv - S. vulgaris* IT Sestriere | *M. silenes-inflatae* | Italy | Sestriere | KY084388 |
| *Mv - S. vulgaris* IT Guarda | *M. silenes-inflatae* | Italy | Guarda | KY084384 |
| *Mv - S. vulgaris* AT St. Johann im Walde | *M. silenes-inflatae* | Austria | St. Johann im Walde | KY084382 |
| *Mv - S. vulgaris* CH Lago Sambuco | *M. silenes-inflatae* | Switzerland | Lago Sambuco | KY084380 |
| *Mv - S. vulgaris* FR Col du Lautaret | *M. silenes-inflatae* | France | Col du Lautaret | KY084383 |
| *Mv - S. vulgaris* FR Galabier | *M. silenes-inflatae* | France | Galabier | KY084381 |
| *Mv - S. vulgaris* IT St. Anna | *M. silenes-inflatae* | Italy | St. Anna | KY084389 |
| *Mv - S. vulgaris* IT Mt. Parrora | *M. silenes-inflatae* | Italy | Mt. Parrora | KY084391 |
| Mv - *S. maritima* IE Inishmore | *M. silenes-inflatae* | Ireland | Inishmore | KY084362 |
| *Mv - S. maritima* IS Stykkisholmur | *M. silenes-inflatae* | Iceland | Stykkisholmur | KY084361 |
| *Mv - S. flos cuculi* UK Great Cumbrae Is. | *M. coronariae* | United Kingdom | Great Cumbrae Is. | KY084325 |
| *Mv - S. campanula* IT Val de Pesio | n/a | Italy | Val de Pesio | KY084347 |
| *Mv - S. caroliniana* US Gilbert Crk | n/a | United States | Gilbert Creek, KY | KY084348 |
| *Mv - S. nutans* CH Guarda | *M. violaceum* | Switzerland | Guarda | KY084368 |
| *Mv - S. nutans* FR Bois Carre | *M. violaceum* | France | Bois Carre | KY084370 |
| *Mv - S. nutans* FR Col du Lautaret | *M. violaceum* | France | Col du Lautaret | KY084369 |
| *Mv - S. vulgaris* FR Bois Carre | *M. silenes-dioicae* | France | Bois Carre | KY084386 |
| *Mv - S. dioica* FR Bois Carrre | *M. silenes-dioicae* | France | Bois Carrre | KY084352 |
| *Mv - S. dioica* FR Bois Freaux | *M. silenes-dioicae* | France | Bois Freaux | KY084353 |
| *Mv - S. dioica* UK Great Cumbrae Is. | *M. silenes-dioicae* | United Kingdom | Great Cumbrae Is. | KY084355 |
| *Mv - S. dioica* UK Priddy | *M. silenes-dioicae* | United Kingdom | Priddy | KY084357 |
| *Mv - S. dioica* IT Val de Pesio | *M. silenes-dioicae* | Italy | Val de Pesio | KY084354 |
| *Mv - S. dioica* UK Charterhouse | *M. silenes-dioicae* | United Kingdom | Charterhouse | KY084356 |
| *Mv - S. dioica* CH Olivone | *M. silenes-dioicae* | Switzerland | Olivone | KY084351 |
| *Mv - S. vulgaris* US Broadway | *M. lychnidis-dioicae* | United States | Broadway | KY084379 |
| *Mv - S. latifolia* US Broadway | *M. lychnidis-dioicae* | United States | Broadway | KY084334 |
| *Mv - S. vulgaris* FR Orsay | *M. lychnidis-dioicae* | France | Orsay | KY084392 |
| *Mv - S. latifolia* FR Orsay | *M. lychnidis-dioicae* | France | Orsay | KY084343 |
| *Mv - S. latifolia* UK Shingle St. | *M. lychnidis-dioicae* | United Kingdom | Shingle St. | KY084346 |
| *Mv - S. latifolia* UK Aldeburgh | *M. lychnidis-dioicae* | United Kingdom | Aldeburgh | KY084345 |
| *Mv - S. latifolia* IT St. Anna | *M. lychnidis-dioicae* | Italy | St. Anna | KY084340 |
| *Mv - S. latifolia* IT St. Anna | *M. lychnidis-dioicae* | Italy | St. Anna | KY084341 |
| *Mv - S. latifolia* IT Carpineti | *M. lychnidis-dioicae* | Italy | Carpineti | KY084335 |
| *Mv - S. vulgaris* IT Lamole | *M. lychnidis-dioicae* | Italy | Lamole | KY084390 |
| *Mv - S. latifolia* IT Lamole | *M. lychnidis-dioicae* | Italy | Lamole | KY084336 |
| *Mv - S. vulgaris* IT San Gimignano | *M. lychnidis-dioicae* | Italy | San Gimignano | KY084393 |
| *Mv - S. latifolia* IT San Gimignano | *M. lychnidis-dioicae* | Italy | San Gimignano | KY084344 |
| *Mv - S. vulgaris* IT Volpaia | *M. lychnidis-dioicae* | Italy | Volpaia | KY084385 |
| *Mv - S. latifolia* IT Volpaia | *M. lychnidis-dioicae* | Italy | Volpaia | KY084337 |
| *Mv - S. vulgaris* IT Montemuro | *M. lychnidis-dioicae* | Italy | Montemuro | KY084387 |
| *Mv - S. latifolia* IT Montemuro | *M. lychnidis-dioicae* | Italy | Montemuro | KY084338 |
| *Mv - S. latifolia* IT Borro | *M. lychnidis-dioicae* | Italy | Borro | KY084339 |
| *Mv - S. latifolia* IT Fornello | *M. lychnidis-dioicae* | Italy | Fornello | KY084342 |
| *Mv - S. douglasii* US Wright’s Lake | n/a | United States | Wright’s Lake, CA | KY084358 |
| *Mv - S. lemmonii* US Wright’s Lake | n/a | United States | Wright’s Lake, CA | KY084359 |
| *Mv - S. acaulis* US Rocky Mtns. | *M. silenes-acaulis* | United States | Rocky Mtns. | KY084330 |
| *Mv - S. acaulis* IT Val de Pesio | *M. silenes-acaulis* | Italy | Val de Pesio | KY084333 |
| *Mv - S. acaulis* FR Orsiera Rocciavre | *M. silenes-acaulis* | France | Orsiera Rocciavre | KY084331 |
| *Mv - S. acaulis* FR Puy Vachier | *M. silenes-acaulis* | France | Puy Vachier | KY084332 |
| *Mv - S. maritima x S. vulgaris* UK Charterhouse | *M. lagerheimii* | United Kingdom | Charterhouse | KY084360 |
| *Mv - S. maritima* UK Charterhouse | *M. lagerheimii* | United Kingdom | Charterhouse | KY084364 |
| *Mv - S. maritima* UK Priddy | *M. lagerheimii* | United Kingdom | Priddy | KY084367 |
| *Mv - S. maritima* UK Aldeburgh | *M. lagerheimii* | United Kingdom | Aldeburgh | KY084363 |
| *Mv - S. vulgaris* CH Bugnei | *M. lagerheimii* | Switzerland | Bugnei | KY084365 |
| *Mv - S. vulgaris* CH Oberalppass | *M. lagerheimii* | Switzerland | Oberalppass | KY084366 |
| *Mv - S. flos jovis* IT Val de Pesio | *M. lagerheimii* | Italy | Val de Pesio | KY084326 |
| *Mv - A rupestre* FR Chambery | *M. lagerheimii* | France | Chambery | KY084375 |
| *Mv - Sap. ocymoides* IT Cesana Tor | *M. saponariae* | Italy | Cesana Tor | KY084394 |
| *Mv - Sap. ocymoides* IT Duc | *M. saponariae* | Italy | Duc | KY084395 |
| *Mv - Sap. officinalis* IT Borro | *M. saponariae* | Italy | Borro | KY084397 |
| *Mv - Sap. officinalis* IT Maggia | *M. saponariae* | Italy | Maggia | KY084398 |
| *Mv - Sap. ocymoides* IT St. Anna | *M. saponariae* | Italy | St. Anna | KY084396 |
| *Mv - Sap. officinalis* FR Vallouise | *M. saponariae* | France | Vallouise | KY084399 |
| *Mv - D. sylvestris* FR La Grave | n/a | France | La Grave | KY084322 |
| *Mv - D. sylvestris* IT Cesana Tor | n/a | Italy | Cesana Tor | KY084323 |
| *Mv - D. carthusianorum* CH Olivone | n/a | Switzerland | Olivone | KY084314 |
| *Mv - D. monspessulanus* FR Chambery | n/a | France | Chambery | KY084317 |
| *Mv - D. pavonius* IT St. Anna | n/a | Italy | St. Anna | KY084318 |
| *Mv - D. pavonius* IT Val de Pesio | n/a | Italy | Val de Pesio | KY084321 |
| *Mv - D. carthusianorum* CH Bugnei | n/a | Switzerland | Bugnei | KY084316 |
| *Mv - D. pavonius* IT St. Anna | n/a | Italy | St. Anna | KY084319 |
| *Mv - D. alpinus* IT Orsiera Rocciavre | n/a | Italy | Orsiera Rocciavre | KY084313 |
| *Mv - D. pavonius* IT St. Anna | n/a | Italy | St. Anna | KY084320 |
| *Mv - D. carthusianorum* CH Lago Sambuco | n/a | Switzerland | Lago Sambuco | KY084315 |
| *Mv - P. saxifraga* AT St. Johann im Walde | *M. dianthorum* | Austria | St. Johann im Walde | KY084327 |
| *Mv - P. saxifraga* IT Saracinesco | *M. dianthorum* | Italy | Saracinesco | KY084328 |
| *Mv - G. repens* CH Grosio | *M. dianthorum* | Switzerland | Grosio | KY084324 |
| *Mv - S. paradoxa* IT Lamole | n/a | Italy | Lamole | KY084371 |
| *Mv - S. paradoxa* IT Montecchiello | n/a | Italy | Montecchiello | KY084372 |
| *Mv - S. paradoxa* IT Santa Vittorina | n/a | Italy | Santa Vittorina | KY084373 |
| *Mv - S. parryi* US Olympics | n/a | United States | Olympics | KY084374 |
| *Mv - S. virginica* US Sugar Hollow | n/a | United States | Sugar Hollow, VA | KY084378 |
| *Mv - S. caroliniana* US Clifton Pond | n/a | United States | Clifton Pond, NC | KY084350 |
| *Mv - S. caroliniana* US Virginia Beach | n/a | United States | Virginia Beach, VA | KY084349 |
| *Mv - S. virginica* US Floyd | n/a | United States | Floyd, VA | KY084377 |
| *Mv - S. virginica* US Ragged Mtn. | n/a | United States | Ragged Mtn., VA | KY084376 |
| *Mv - Pers. bistorta* FR Bois Carre | *M. bistortarum* | France | Bois Carre | KY084329 |
